# A 10-year retrospective observational study on the utility and prescription standards of dexamethasone in pediatric neuro-oncosurgery in a tertiary care center

**DOI:** 10.1007/s00381-022-05569-6

**Published:** 2022-06-08

**Authors:** Anutra Chumbala Na Ayudhaya, Scott R. Morrison, Chandrasekaran Kaliaperumal, Pasquale Gallo

**Affiliations:** 1 The University of Edinburgh Medical School, Edinburgh, Scotland; 2 NHS Lothian, Edinburgh, Scotland; 3 Consultant Adult and Paediatric Neurosurgeon, Department of Clinical Neuroscience, NHS Lothian, Edinburgh, Scotland

**Keywords:** Dexamethasone, Pediatric, Neurosurgery, Oncology, CNS tumors, Guidelines, Standardization

## Abstract

**Object:**

This study aimed to retrospectively assess dexamethasone utility in pediatric CNS tumor patients over a 10-year period, to better understand dosing variability, and highlight optimal practice.

**Methods:**

All pediatric CNS tumor cases managed operatively for a 10-year period at a single center were reviewed. Information was gathered on demographics, dexamethasone doses, course durations, weaning regimes, PPI co-prescription, adverse events, and route of administration. Comparison within these groups was analyzed through use of statistical testing.

**Results:**

One hundred twenty-seven patients received 193 dexamethasone courses. Median age was 7 years, with a median weight of 27.9 kg. Most common tumor type was astrocytoma (24.8%). Median daily dose was 8 mg, with twice-daily dosing most common. Median course duration was 8 days, ranging from 1 to 1103 days. Median weaning duration was 11.5 days. Daily dose was not correlated with patient weight and the median daily dose per kg was 0.2319 mg/kg. Incidence of adverse effects was 14.5% across all course lengths, with weight gain most common. The short-term course duration (<14 days) had the lowest adverse event incidence, with direct correlation between course length and adverse effect incidence. Dexamethasone dose per kg was not significantly different between patients with and without adverse effects. No relationship was noted between adverse effects incidence and administration route (intravenous compared to oral). 64.2% of patients received concurrent PPI with 35.8% receiving no PPI, with 1 gastrointestinal side effect noted in the PPI-receiving population.

**Conclusions:**

Large variation was seen in practice, with prescriptions appearing based on clinician preference and symptom severity rather than patient age or weight. Future guidelines should consider lower dose regimens than are currently presented with less frequent dosing as these may benefit quality of life. Weaning period can be relatively rapid for most patients, taking place in 2–3 days. PPI co-prescription does not seem to add significant benefit. We recommend using a standardized guideline of 0.2 mg/kg/day (max 8 mg/day) given OD or BD, with PPI cover where necessary. For acute presentations, we recommend limiting dexamethasone treatment to <14 days. These recommendations can be adjusted for individual cases to yield optimal results.

## Introduction

Central nervous system (CNS) tumors are the most common solid tumor in the pediatric population and the second commonest cause of cancer in patients 0–19 years of age [1–3]. These tumors unfortunately still represent the most common cause of cancer-related death in children, with a 5-year mortality of 30% [1, 4].

CNS tumors have heterogeneous symptomatology due to raised intracranial pressure exerted by the mass effect of the tumor, and tumor-related vasogenic edema [5, 6].

Neurosurgical tumor resection represents the primary management modality for curative treatment [7]. However, tumor resection is not always possible. For these patients, and for those awaiting surgery, effective symptomatic treatment is vital. Corticosteroids have been the cornerstone treatment for symptomatic management in cerebral tumors since their benefits were first noted in 1957 [8]. Dexamethasone is the principal corticosteroid in this role [9, 10]. Dexamethasone’s benefits are not purely symptomatic and have been shown in certain cases to extend lifespan [11]. However, dexamethasone use comes with numerous side effects, including Cushing’s syndrome, gastrointestinal (GI) problems, anxiety, mood disorders, and bone formation disruption [12–15].

These side effects are reported to be dose-dependent in both severity and frequency [9, 16, 17]. Furthermore, dexamethasone has to be carefully weaned to prevent acute adrenal insufficiency crisis or withdrawal syndromes [18]. Dosing recommendations for adults have been proposed [9, 19]. However, there is a paucity of evidence regarding appropriate prescription practices for children, with the BNFc only advising in life-threatening cerebral edema [19–21].

Dexamethasone dosing is therefore currently guided by the patients’ clinical factors and physician judgement, resulting in disparities in treatment regimens between individual physicians and various centers [20, 21]. Guidelines for dexamethasone prescription in pediatric CNS tumor patients would hopefully allow expedited symptomatic control, with optimal dosing, balancing benefit and risk. Our team and colleagues internationally have recognized the paucity of evidence [22].

The aim was to perform a retrospective observational study assessing dexamethasone utility in pediatric CNS tumor patients in a tertiary care center over a 10-year period, to better understand dosing variability, and possibly highlight optimal dosing regimens.

## Methods

Patients were identified from a database of all pediatric patients undergoing CNS tumor-related neurosurgical procedures at a tertiary center within the UK between January 2011 and December 2020.

Data for each patient was retrieved via a local electronic patient record system. Demographic factors of age, sex, weight, and tumor type were assessed. For data analysis, patients were divided into 4 age groups (0–4, 5–9,10–14, 15+ years).

Dexamethasone doses and daily regimens were collected. Where doses had been varied, the dosing regimen maintained for the longest period was used.

Dose duration was calculated from recorded dexamethasone start and end dates. Weaning regimen duration and routes of administration were also reviewed.

Adverse effects reported to be directly or likely associated with dexamethasone were reviewed and categorized.

PPI co-prescription for GI side effect mitigation was also assessed for drug, dosing, and route of administration.

Statistical significance was defined with a confidence interval of 95% (*p* < 0.05). All analyses were carried out on SPSS. Outliers were included in the analysis but not represented on graphs and figures for data presentation.

## Results

The total number of patients reviewed was 164. One hundred and one (61.6%) were male and 63 (38.4%) were female. Thirty-seven (22.6%) patients did not receive dexamethasone and were excluded. One hundred twenty-seven (77.4%) patients received 193 courses of dexamethasone. Eighty-seven (53.0%) patients received 1 course, 25 (15.2%) received 2, 8 (4.9%) patients received 3 courses, while 7 (4.3%) patients had 4 or more courses. The median number of dexamethasone courses received was 1 [1, 1] (*n* = 164).

The median age of patients receiving dexamethasone was 7 years with a median weight of 27.9 kg. The most common tumor types were astrocytomas (24.8%), medulloblastomas (11.3%), and ependymomas (10.0%).

The median dexamethasone dose per single administration was 4 mg. The most common dosing regimen was twice daily (BD) making up 74.8%; the second most common was three times daily (TDS) at 12.6%, followed by once daily (OD) at 8.7%, 4 times daily (QDS) with 3.1%, and 8 times daily with 0.8%.

**Fig. 1:**
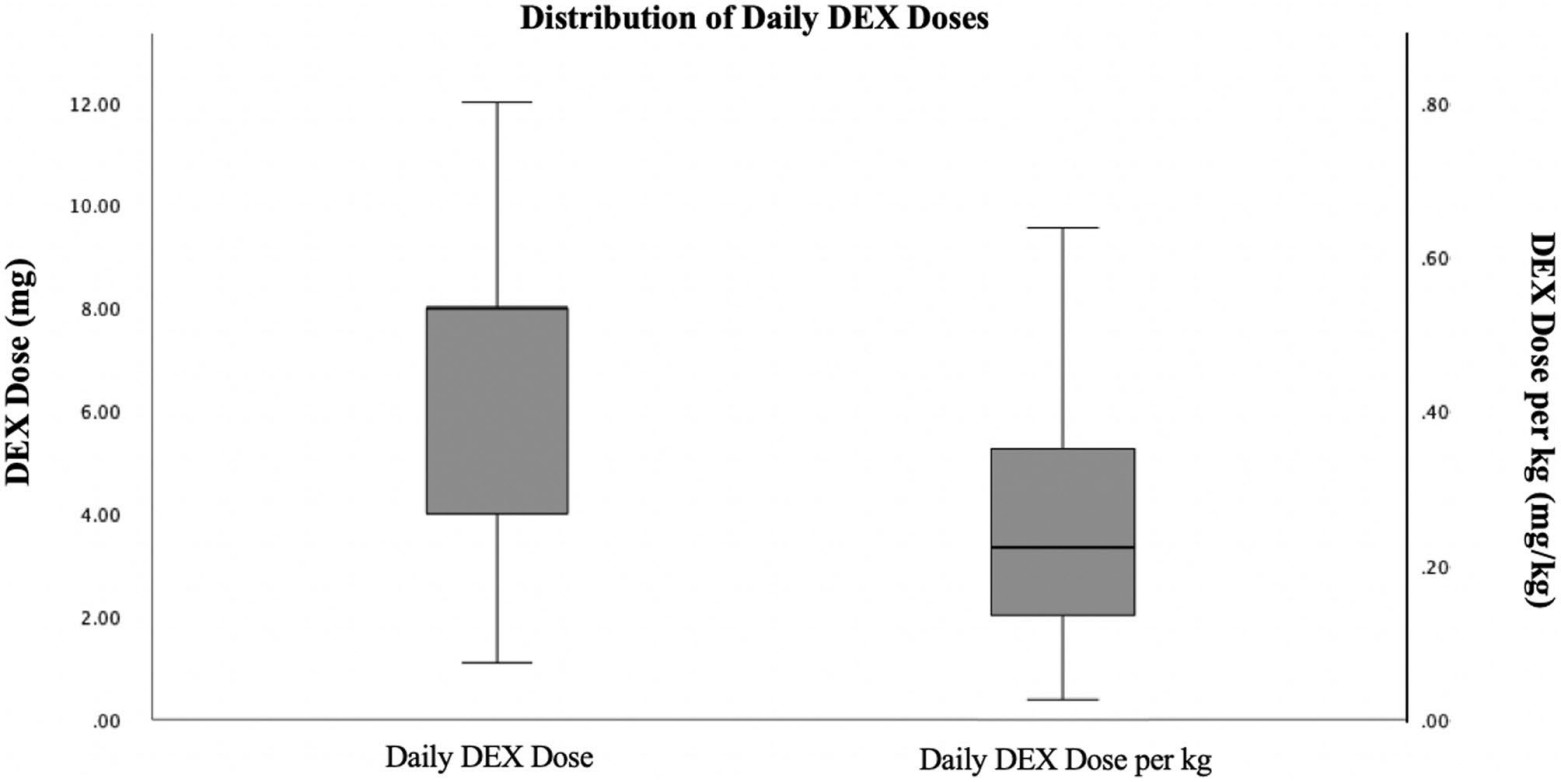
Box plot representing the distribution of daily dexamethasone (DEX) doses and daily DEX dose per kg given for all patients. Daily DEX dose *n* = 126. Daily DEX dose per kg *n* = 115 (weight unable to be assessed in 11 cases). Outliers were not represented within this graphic. Outliers in Daily DEX Dose category = 16, 16, 16, 16, 16.5, 26.4. Outliers in Daily DEX Per KG category = 0.7186, 0.7273, 0.8000, 0.9756, 1.2500, 1.6604

The median daily dose was 8 mg, ranging from 1.1 to 26.4 mg (Fig. 1). The daily dose was not seen to be correlated with patient weight. Median daily dose per kg was 0.232 mg/kg and ranged from 0.026 to 1.664 mg/kg (Fig. 1).

The median daily dose per kg for males and females were 0.232 mg/kg and 0.243 mg/kg respectively.

**Fig. 2:**
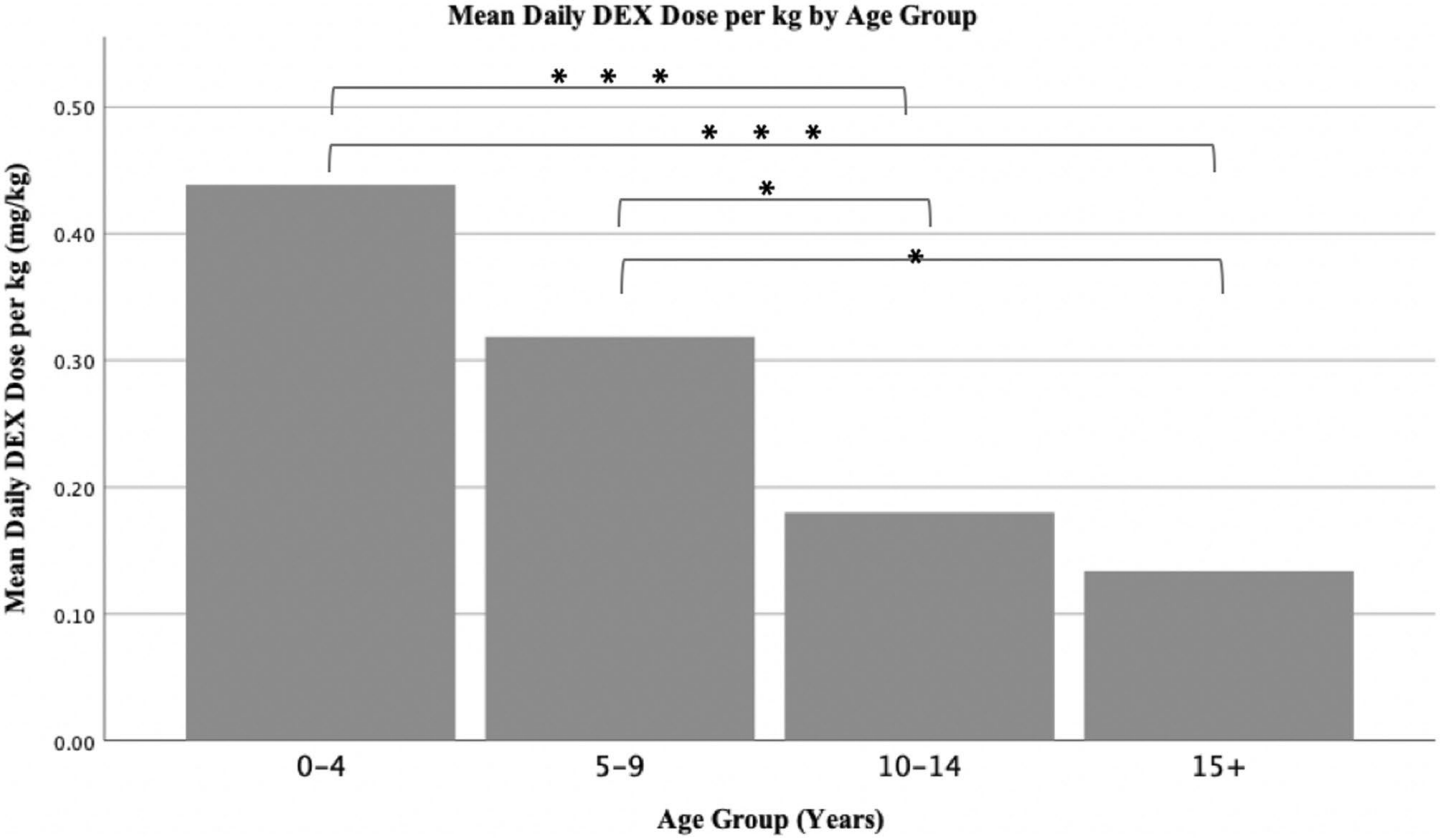
Bar chart representing the average daily dexamethasone (DEX) dose per kg received across different age groups. One-way ANOVA (*F*(7,76) = 3.187, *p* = 0.05) was utilized to show significant differences between groups. Post-hoc Tukey test was utilized to highlight specific significant differences, represented by *p < 0.05, and ***p < 0.005

Dexamethasone dose per kg was significantly inversely correlated with age (*p* < 0.01). The age group that averaged the highest dexamethasone dose per kg was 0–4 years. This was followed by the 5–9 age group, then 10–14, and 15+ (Fig. 2). Differences between age groups were statistically significant as determined by one-way ANOVA (*F*(7,76) = 3.187, *p* = 0.05).

The median dexamethasone dose duration was 8 days, ranging from 1 to 1103 days. The most common duration is 3 days. No obvious trends in dose duration were noted across age groups.

44.9% of patients received intravenous (IV) dexamethasone only. 32.7% received oral (PO) dexamethasone only. 11.2% initially received IV dexamethasone then switched to PO. The remaining 11.2% received a complex regimen, alternating frequently between IV, PO, and nasogastric (NG) routes.

**Fig. 3:**
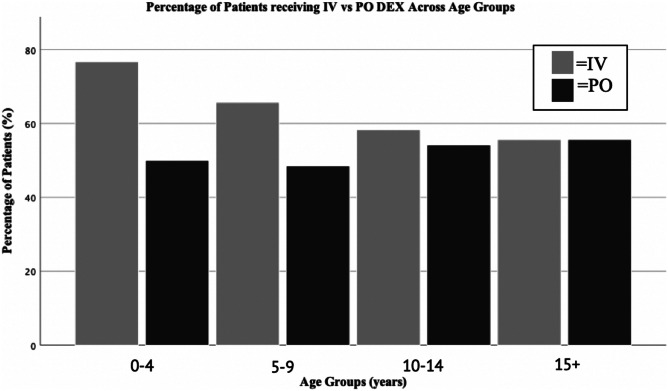
Comparative bar chart representing intravenous versus oral administration routes of dexamethasone (DEX) between various age groups. Number of patients where route was able to be assessed = 98. This chart records route utilized at any time in treatment, and therefore total percentage (IV% + PO%) for each group can be > 100%

IV dexamethasone was most commonly used in young age groups and less common in older patients. In the 0–4 age group, 76.7% received IV dexamethasone as part of their administrative regimen. In the 5–9 age group, 65.7% received IV dexamethasone. In the 10–14 age group, 58.3%, and in the 15+ age group, 55.6% received IV dexamethasone during their course of treatment. PO dexamethasone was used more in older age groups but was used relatively more consistently throughout all age groups (Fig. 3).

The median weaning regimen duration was 11.5 days, ranging from 2 to 129 days. The most common weaning period was 2 days.

**Fig. 4:**
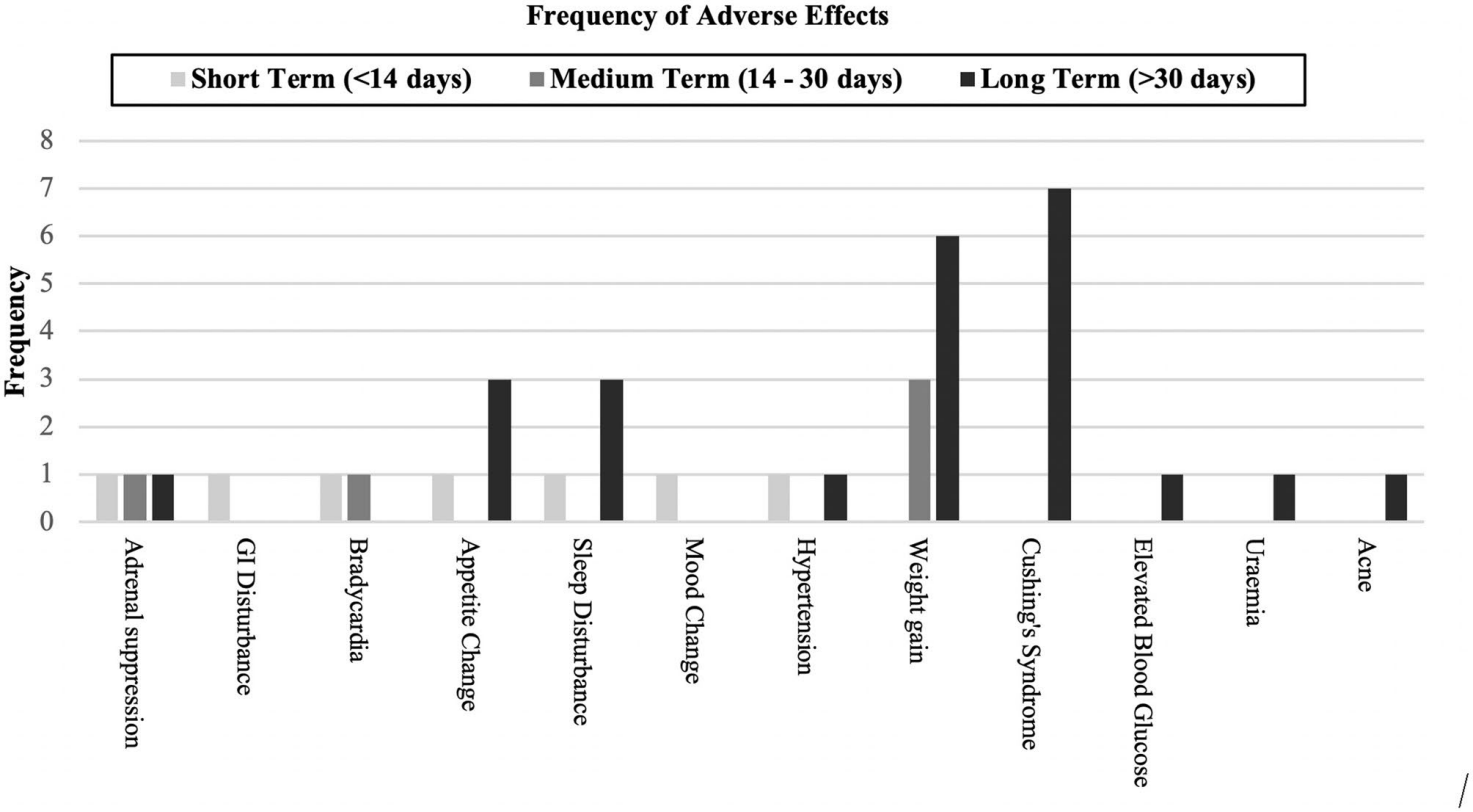
Bar chart representing the full number of courses of dexamethasone (DEX), and the frequency of associated adverse effects subdivided by treatment course length. Number of courses reviewed = 193. In cases where one patient experienced >1 adverse effect, both of these were recorded (i.e., Cushing’s syndrome and adrenal suppression)

The overall incidence of adverse effects was 19.7% with weight gain (4.7%) being the most common. The frequency of adverse effects was directly related to the length of the course (Fig. 4).

Independent-samples *T*-test revealed the mean daily dexamethasone doses per kg were not significantly different between patients with and without adverse effects (*t*(113) =  −114, *p* = 0.909). However, average dexamethasone course durations were significantly different between patients with and without adverse effects (*t*(123) = 2.486, *p* = 0.014). The data highlighted course length was directly related to the incidence of adverse effects, with 6.3% of the short-term (<14 days) course length patients experiencing adverse effects, 23.5% of medium-term (14–30 days), and 44.8% of long-term (>30 days) patients. These groups were independently assessed with regard to dose and it was seen that the short-term mean was 0.319 mg/kg, for the medium-term it was 0.254 mg/kg, and for long-term it was 0.237 mg/kg.

The age group with the highest incidence of adverse effects was 15+ at 23.8%. The 0–4 age group experienced the least side effects at 11.1%. However, the relationship between age and incidence of adverse effects was not significant (*p* = 0.580).

There was no significant relationship between adverse effects and administration route.

**Fig. 5:**
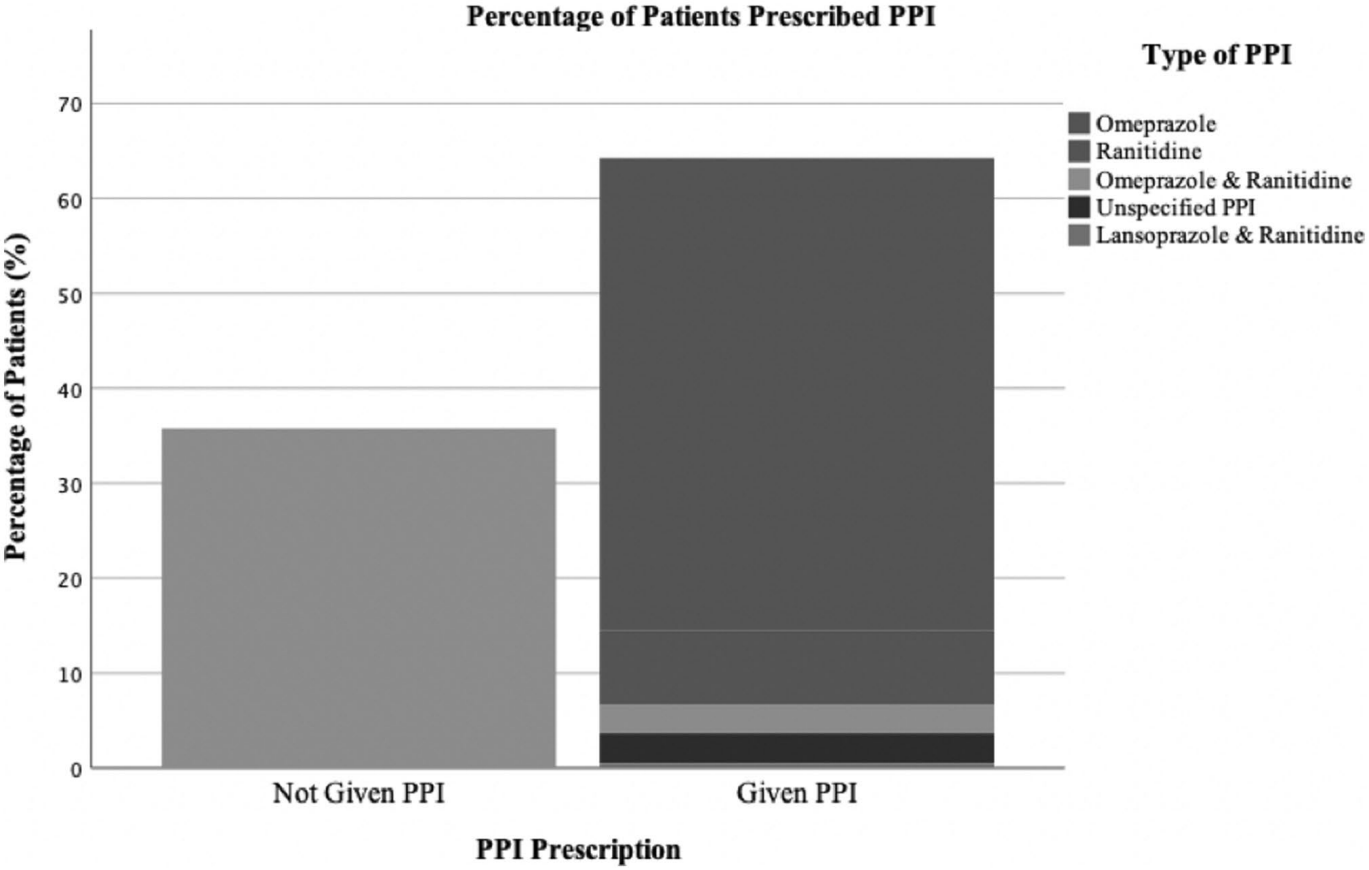
Proportional bar chart representing the full number of courses of dexamethasone (DEX), and whether a concurrent PPI was prescribed. Number of courses reviewed = 193. Number given PPI = 124 (64.2%). Number not given = 69 (35.8%). Those given PPI were subdivided by PPI utilized. Unspecified PPI noted when PPI use recorded, but specific agent unable to be assessed, with number unspecified = 6

64.2% of patients received PPI during their dexamethasone treatment with 35.8% not receiving any PPI. 49.7% received omeprazole. 7.8% received ranitidine. 3.1% received both omeprazole and ranitidine. 0.5% received lansoprazole and ranitidine and 3.1% received an unspecified PPI (Fig. 5).

The most common and median omeprazole dose was 20 mg. No patients in the non-PPI group experienced GI side effects, with 1 patient in the PPI group experiencing GI side effects.

## Discussion

There seems to be a preference for a 4 mg BD dexamethasone prescription which is in line with some of the guidelines proposed for adults [10, 23]. Regardless, the range of daily doses was wide (1.1–26.4 mg). When adjusted for weight, this wide range was still noted (0.0260–1.664 mg/kg). This may be attributed to heterogeneous symptom severity that this study was unable to assess. It should be noted that this study did not assess objective symptom relief from different doses of dexamethasone; however, in any case where the initial dose was unsatisfactory in utility, the increased dose that was seen to be effective was recorded, allowing a recording of the dose at which patients adequately responded to treatment.

Doses per kg for male and female patients were not significantly different, suggesting patient sex was not significant in determining dosing.

There was no correlation between patient weight and daily dexamethasone dose; however, dexamethasone dose per kg was negatively correlated with age, implying younger patients received higher doses relative to their body weight. This could reflect clinicians prescribing fixed doses to patients of varying weights. This may be further evidenced by the fact that no correlation was noted between patient weight and daily dexamethasone dose.

These findings suggest dosing decisions may be based on clinician preference, and symptom severity, overlooking or taking into minor consideration patient weight, age, and sex. It was, however, seen that different tumor types received different dexamethasone doses for symptomatic relief, which may warrant further investigation.

The BNFc published recommendations for dexamethasone prescription for children, however, it only concerns patients with life-threatening cerebral edema [19]. It is the only guideline in wide circulation that offers any guide toward dosing of dexamethasone in these cases. It recommends an initial dose of 16.7 mg and a maintenance dose of 26.4 mg daily for 3 days for those <35 kg, and for those >35 kg, an initial dose of 20.8 mg and a maintenance regime of 39.6 mg daily for 3 days. Even though dexamethasone prescribed in this center was primarily for acutely non-life-threatening cases of cerebral edema, the comparison was of interest. The mean doses in this study were significantly lower than recommendations for both <35 kg (*p* < 0.001) and >35 kg (*p* < 0.01). For those <35 kg, the average doses were 73.9% (*n* = 69) lower than the recommendation. For patients >35 kg, average doses were 80.6% (*n* = 46) lower. This is concordant with literature from the adult population, claiming lower doses of dexamethasone may be sufficient for symptomatic management of CNS tumors [10, 14, 24]. Literature also suggests less frequent dosing regimens—such as BD—may be associated with better sleep and improved quality of life, which is especially pertinent in a population where maximizing quality of life is of paramount importance [23].

There was a large variation in dexamethasone dose duration (1–1103 days). This range had a median value of 8 days, with the most common value being 3 days. This highlighted that most cases have dose durations <30 days, and those extending to the extremes of the range represent outliers who were post-operatively managed with dexamethasone for extended periods (often in palliative settings).

The most common routes of administration were IV and PO. IV routes were more commonly used for younger patients, likely illustrating the inability of younger patients to tolerate oral medications. There was no association between adverse effects and route of administration.

A wide range of weaning regimens were recorded (2–129 days). Long tapering periods were mainly used in complex cases where dose reduction risked symptom recurrence. The most common tapering regimen was 2 days (all dexamethasone weaned over 2 days). This seems acceptable, with no serious adverse events reported. However, adult studies have shown the risk of adrenal insufficiency to be as high as 48.7% upon discontinuation of oral corticosteroids [25]. Further studies addressing this will be crucial to gain insight into ideal tapering schemes. Adult guideline recommendations have suggested a tapering regimen over a period of 2 weeks which can be extended for symptoms [10]. However, as noted in our data, courses less than 2 weeks provide optimal results with regards to incidence of adverse effects, and no weaning-related adverse effects were noted in the 2-day group, suggesting shorter weaning regimes may well be favorable in children.

No life-threatening adverse events related to dexamethasone were reported. The side effects observed were in keeping with common adverse effects reported in the literature, with weight gain being the most common [14, 23]. Although, the incidence of these was lower than those reported by other authors [12, 26].

The mean dexamethasone dose per kg for patients with and without adverse effects was not significantly different (*P* = 0.909), implying the incidence of adverse effects was not dose-dependent, contrasting existing literature [9, 10, 16, 17]. Recording the incidence of adverse effects that allows for severity analysis in the future would enable a more accurate assessment of correlation.

The mean dose durations for patients with and without adverse effects were significantly different (*p* = 0.014), showing the incidence of adverse effects to be dependent on dose duration. This agrees with existing literature, and further highlights the importance of minimizing course duration and redundant dexamethasone prescription [12, 14, 24, 27]. The group receiving <14 days of dexamethasone (including the weaning period) had, by far, the lowest incidence of adverse effects. This was assessed in the context of dose and it was seen that the patient group on short-term (<14 days) courses of dexamethasone received the highest dose/kg. This seems to further highlight that the length of the course is more significant in the context of adverse events than the dose (especially in those using dexamethasone for <31 days).

Despite older patients receiving lower dexamethasone doses per kg, the incidence of adverse effects was higher, likely reflecting the ability of older patients to better report side effects. This should be taken account of, as it highlights the importance of screening adverse events in younger patients.

There was no significant relationship between the incidence of adverse effects and the route of dexamethasone administration. Appearing to highlight that the ideal route of administration would be one that the patient tolerates best. In the future, it would be useful to assess if different routes of administration are associated with greater symptomatic relief.

Current guidelines in adults do not advise PPI co-prescription [28, 29]. Some authors suggest routine co-prescription is not required due to the low incidence of GI side effects (0.4–1.8%) [30]. Many adverse effects of PPIs on the gut microbiome, immune function, and bone fractures have been noted in the pediatric population; however, these are relatively rare [31]. In this study, PPIs were co-prescribed to 64.2% of patients. The low incidence of GI side effects in this patient group is positive, but with the only GI side effect identified in the patient group prescribed PPIs, it does appear to agree with the adult guidelines. Further large-scale studies would be ideal to assess the value of PPIs in these patients. It should be noted that a small group of patients were prescribed ranitidine which has been withdrawn under advice from the Royal College of Pediatrics and Child Health (RCPCH) due to the presence of NMDA [32].

One study highlighted 80% of sampled institutions in Canada lacked formal guidelines, leading to large discrepancies in prescription practices, and this lack of guidelines has been noted elsewhere for many years [20, 21]. Lack of standardization between centers and clinicians may be leading to variable time to effective treatment, increased risk of adverse effects, and suboptimal symptom control. Formulation of standardized guidelines would represent a step toward more reproducible, easily understood, and safe pediatric CNS tumor management.

Current recommendations from BNFc only reference life-threatening cases of cerebral edema, and moreover, they divide a continuous spectrum of patients into only two groups based on weight. Reviewing existing evidence, and findings from this study, it seems any guidelines developed in the future should consider the following:

1. Significantly lower doses are seen to be acceptable for use across all age groups, with our initial recommendation being to utilize 0.2 mg/kg/day, up to 8 mg/day, OD or BD depending on local preference. This is based on these doses providing adequate symptom relief in most patients, and (although our data did not show this) reducing any risk which may exist from higher dose regimes. These dosing regimens will require further investigation to ensure optimal dosing regimens, and of course some adjustments will be necessary in certain cases.
2. As has been highlighted in other studies, the length of the dexamethasone course was significant in determining the risk of adverse effects. We would advise—where possible—to utilize regimes under 14 days in length (inclusive of the weaning regime—which, if using the doses recommended above, should be 2–3 days in length). The weaning period advised here also fits with published literature on rapid weaning regimens which can be very effective with close monitoring [9].
3. The route of administration had no effect on adverse effects, and the optimal route of administration should be utilized on a patient-by-patient basis.
4. Based on the data in this study, the benefits of PPIs are unclear. The wider literature also demonstrates little benefit in reducing the risk of steroid gastropathy [33]. Therefore, PPI co-prescription may not always be routinely required. For those experiencing gastrointestinal side effects, prescription of PPI according to local drug policy would be acceptable. As per RCPCH guidelines, ranitidine should be avoided.
5. In cases where longer courses of administration are advisable, the same dosing recommendations would be advised initially, but reaching the lowest effective dose for extended periods would be optimal.

This study highlighted route of dexamethasone administration appears to have no effect on the incidence of adverse effects. Removing concern that the increased bioavailability of IV dexamethasone versus oral preparations may increase the risk of adverse effects [34]. This result does require further validation.

For a number of patients, certain information was unobtainable. This limited inclusion of patients in data analysis. Due to the retrospective nature of this study, certain data points were unable to be assessed due to a lack of formal data collection in specific areas. For example, adverse effects were treated as categorical variables, and the severity of these side effects was not assessed. Furthermore, the degree of symptomatic relief achieved and its effects on patient outcomes could not be assessed.

A multi-center prospective observational study would provide higher quality evidence with a broader scope of analysis and would also allow evidenced guideline development, through discussion between multiple clinicians in both neurosurgery and neuro-oncology.

## Conclusions

Dexamethasone was well tolerated and the doses used in this center were considerably lower than the doses recommended by the BNFC. Prescribing practices have been based on clinician preference and symptom severity rather on patient age or weight. Incidence of dexamethasone-associated adverse effects was related to dexamethasone course duration. PPI co-prescription does not seem to add significant benefits. The findings of this study highlight variability in practice and the necessity of implementing uniform dosing protocols to standardize treatment.

Given the heterogeneous nature of pediatric neuro-oncology, individually tailored therapy regimes may be necessary. As a starting point, we recommend using a standardized guideline of 0.2 mg/kg/day (max 8 mg/day) given OD or BD, with PPI cover where necessary. For acute presentations, we recommend limiting dexamethasone treatment to < 14 days. These recommendations can be adjusted for individual cases to yield optimal results.
